# On the Cranial Nerves

**DOI:** 10.3390/neurosci5010002

**Published:** 2023-12-28

**Authors:** Hugo M. Libreros-Jiménez, Jorge Manzo, Fausto Rojas-Durán, Gonzalo E. Aranda-Abreu, Luis I. García-Hernández, Genaro A. Coria-Ávila, Deissy Herrera-Covarrubias, César A. Pérez-Estudillo, María Rebeca Toledo-Cárdenas, María Elena Hernández-Aguilar

**Affiliations:** 1 Doctorado en Investigaciones Cerebrales, Universidad Veracruzana, Xalapa 91190, Mexico; hlibreros@uv.mx; 2 Instituto de Investigaciones Cerebrales, Universidad Veracruzana, Xalapa 91190, Mexico; jmanzo@uv.mx (J.M.); frojas@uv.mx (F.R.-D.); garanda@uv.mx (G.E.A.-A.); luisgarcia@uv.mx (L.I.G.-H.); gcoria@uv.mx (G.A.C.-Á.); dherrera@uv.mx (D.H.-C.); cesperez@uv.mx (C.A.P.-E.); rtoledo@uv.mx (M.R.T.-C.)

**Keywords:** vision, hearing, tasting, smelling, head, face, brain stem

## Abstract

The twelve cranial nerves play a crucial role in the nervous system, orchestrating a myriad of functions vital for our everyday life. These nerves are each specialized for particular tasks. Cranial nerve I, known as the olfactory nerve, is responsible for our sense of smell, allowing us to perceive and distinguish various scents. Cranial nerve II, or the optic nerve, is dedicated to vision, transmitting visual information from the eyes to the brain. Eye movements are governed by cranial nerves III, IV, and VI, ensuring our ability to track objects and focus. Cranial nerve V controls facial sensations and jaw movements, while cranial nerve VII, the facial nerve, facilitates facial expressions and taste perception. Cranial nerve VIII, or the vestibulocochlear nerve, plays a critical role in hearing and balance. Cranial nerve IX, the glossopharyngeal nerve, affects throat sensations and taste perception. Cranial nerve X, the vagus nerve, is a far-reaching nerve, influencing numerous internal organs, such as the heart, lungs, and digestive system. Cranial nerve XI, the accessory nerve, is responsible for neck muscle control, contributing to head movements. Finally, cranial nerve XII, the hypoglossal nerve, manages tongue movements, essential for speaking, swallowing, and breathing. Understanding these cranial nerves is fundamental in comprehending the intricate workings of our nervous system and the functions that sustain our daily lives.

## 1. Introduction

The cranial nerves, comprising a set of twelve paired peripheral nerves, serve as conduits of information between the central nervous system and various regions of the head, neck, and upper torso. Emerging directly from the brain or the brainstem, these nerves are critical mediators of both motor and sensory functions, and they are integral for a multitude of physiological processes, including vision, olfaction, audition, and complex facial movements. Unlike spinal nerves, which emanate from segments of the spinal cord, cranial nerves have a direct connection to specific brain regions, thereby forming a crucial interface for the transmission of specialized sensory and motor signals. From a clinical perspective, understanding the anatomy, functional roles, and pathways of cranial nerves is indispensable for the diagnosis and treatment of various neurological conditions, including cranial nerve neuropathies and certain types of facial pain syndromes. Therefore, the study of cranial nerves has significant implications for scientific research and for medical practice and healthcare outcomes. This review aims to provide an in-depth exploration of the cranial nerves, elucidating their anatomy, functions, and relevance in both health and disease. Briefly, an introductory summary of each nerve name and type is depicted in Table 1.

## 2. Cranial Nerve I: Olfactory Nerve

The first cranial nerve or olfactory nerve is crucial to regulate this sensory system. All mammalian species possess an olfactory sensory system that enables the detection of environmental odors. The efficacy of odor detection is influenced by the composition of gases, vapors, and particulates in the environment. The prevalence of each constituent determines the type of odor perceived [1,2]. Odor detection occurs because the sensed particle is volatile and becomes hydrated within the olfactory epithelium, rendering it chemically active. The sensitivity of odor detection varies across non-human mammalian species and is generally superior to that in humans. Specifically, the human olfactory epithelium has an area of 2 cm^2^, a thickness of 70 µm, and approximately 6 million receptors. In contrast, the canine olfactory epithelium spans around 100 cm^2^ and contains nearly 200 million receptors [3,4]. A highly organized anatomical framework exists to facilitate odor perception, beginning with the nostrils, which funnel environmental air containing odorant particles into the nasal cavity. This cavity houses olfactory receptors, which are bipolar nerve cells that form the olfactory epithelium [5]. These receptor cells have dendrites positioned on the epithelial surface and axons that constitute the olfactory nerve. These axons proceed toward the main olfactory bulb, forming clusters of neurons known as glomeruli [6]. Each glomerulus receives input from thousands of different olfactory cells that contain the same type of receptor molecule, facilitating the initial level of synaptic processing. Glomeruli play a critical role in olfactory information translation by classifying various types of odorants [7].

The subsequent level of odor processing occurs in the piriform cortex, which is composed of astrocytes, interneurons, and primarily the dendrites of mitral and tufted cells. Synaptic interactions between the axons of olfactory receptor cells and the dendrites of mitral and tufted cells occur in the glomeruli. These second-order neurons form synapses with various types of interneurons or with GABAergic granule cells [8]. This establishes reverberating circuits for both negative and positive feedback loops. The axons of the mitral and tufted cells join to form the olfactory tract, conveying information ipsilaterally to various brain areas, including the anterior olfactory nucleus, olfactory tubercle, piriform cortex, amygdala, and entorhinal cortex [9]. The olfactory system is unique in its potential for recovery following an injury to the olfactory bulb, especially when such injury occurs during early developmental stages [10]. The shape of the olfactory bulb is correlated with olfactory function, irrespective of age, gender, and volume. However, its shape undergoes changes with age, which are associated with olfactory disorders [11].

In rodents and some other mammals, the vomeronasal (VNO) or Jacobson’s organ serves as an auxiliary olfactory organ. Unlike the main olfactory system, it is anatomically distinct and specializes in the detection of non-volatile pheromones [12]. In humans, this organ is present, but its detection of pheromones is not consciously perceived, although it does influence sexual behavior [13,14,15].

### 2.1. Pathophysiological Conditions

While traditionally associated with pheromone detection, the human VNO has also been implicated in various pathophysiological conditions. One such condition is the septi nasi sinus, a developmental anomaly characterized by a sinus or cystic structure within the nasal septum. This anomaly can potentially lead to nasal obstruction and subsequent breathing difficulties. Additionally, the VNO’s potential role in detecting stress-related pheromones has been explored in relation to post-traumatic stress disorder (PTSD), a psychiatric condition triggered by experiencing or witnessing traumatic events. While the exact mechanisms remain unclear, alterations in pheromone processing might influence the onset or severity of PTSD symptoms. Furthermore, the ectopic olfactory esthesioneuroblastoma, a rare malignant tumor arising from the olfactory epithelium, can disrupt the normal detection of smells, leading to anosmia. Anosmia, the loss of the sense of smell, not only diminishes the quality of life but can also pose safety risks, such as the inability to detect hazardous odors, such as smoke or gas leaks [12,15].

### 2.2. Virus and Toxins

On the other hand, the olfactory nerve provides a direct conduit from the nasal cavity to the brain, bypassing the blood–brain barrier. This unique anatomical pathway allows for the direct impact of external agents on the central nervous system. Viruses such as the herpes simplex virus and certain neurotoxins exploit this route to enter the brain, potentially leading to infections or neurological damage. However, on the therapeutic front, such a pathway offers a promising route for drug delivery to the brain. This is particularly advantageous for treating central nervous system disorders, as the blood–brain barrier typically hinders the passage of therapeutic compounds. By leveraging the olfactory nerve’s direct access to the brain, researchers are investigating novel strategies to deliver drugs effectively to target areas, potentially opening new avenues for treating neurodegenerative diseases and brain tumors [12,15].

## 3. Cranial Nerve II: Optic Nerve

Vision serves as one of the quintessential sensory modalities in humans, facilitating not only environmental awareness but also muscular coordination for spatial navigation. The scope of information obtained from the external world through the visual apparatus is extensive. The human optical system facilitates the perception of the environment in three dimensions; discerns color, motion, and form; and detects subtle variations, such as depth and spatial relationships with other objects.

The visual process initiates with the capture of light, which is modulated in terms of color and object shape to formulate the phenomenon commonly referred to as “seeing”. Initially, light traverses the cornea, a highly differentiated and transparent tissue specialized for the refraction and transmission of light [16]. As the outermost layer of the eye, the cornea is in perpetual contact with the environment and overlays other ocular structures, such as the iris, pupil, and anterior chamber [17]. Boasting a refractive power of 43 diopters, the cornea represents the epithelial tissue contributing most significantly to the eye’s focusing capability. This focusing capacity is primarily attributed to the interface created between the tear film and the cornea, the latter of which also has a high density of sensory nerve endings originating from the trigeminal nerve.

Upon passing through the cornea, light impinges on the central aperture of the iris, commonly known as the pupil, which serves as a conduit between the anterior and posterior chambers of the eye [18]. The muscle governing the iris adjusts the amount of light striking the pupil through a network of smooth circular and radial muscle fibers. These fibers contract to either constrict or dilate the pupil in response to variations in light intensity [19,20]. This contractile modulation is orchestrated by the pretectal olivary nucleus, which sends projections to postganglionic fibers that release acetylcholine, thereby activating the iris sphincter muscle and inducing pupil contraction. Pupil constriction occurs through two distinct mechanisms: (1) the parasympathetic innervation of the sphincter muscle triggered by sympathetic pathway activation leads to muscle relaxation, and (2) the excitation of the alpha-1 adrenergic pathway results in the contraction of the dilator muscle. Both processes necessitate changes in calcium concentration for effective modulation [21,22,23,24,25].

After its interaction with the iris, light proceeds to the lens, a structure that shares several attributes with the cornea, such as transparency, colorlessness, biconvexity, flexibility, and avascularity [26,27]. Positioned posterior to the iris and vitreous humor and anterior to the vitreous body, the lens owes its transparency to the highly ordered arrangement of its constituent cells, known as fibers, and the extracellular matrix [28]. Such a matrix is confined within the lens capsule, while the fibers—composed of crystallin proteins—form a syncytium, functioning as a singular unit that intercommunicates and absorbs short wavelengths. Remarkably, the lens serves as an absolute barrier to wavelengths shorter than 300 nm [29,30]. The primary function of the lens is to focus incoming rays to form a coherent image on the retina, regardless of the object’s distance. This is enabled by the lens’s variable refractive index, which arises from a central concentration of crystallin proteins that gradually diminishes towards the outer layers. In addition, the lens achieves this focusing ability through alterations in its curvature and thickness, a phenomenon referred to as “accommodation”. The degree of lens accommodation is one of the metrics utilized by the brain in estimating the distance of an observed object [31,32,33,34,35].

Following its passage through the lens, light waves traverse the vitreous humor to finally converge upon the retina. The retina is a complex structure comprising ten distinct layers, and it serves as a highly specialized and stratified tissue designed for the efficient transduction of optical signals into neural impulses, thereby facilitating the perception of the visual environment. The layers categorized from the most superficial to the innermost regions are summarized as follows [36,37]:

1. Pigmented Epithelium: This layer contains melanin, accounting for its coloration.
2. Photoreceptor Cell Layer: This is the location of cones and rods, the sensory cells responsible for detecting light.
3. Outer Limiting Membrane: This layer is formed by adherent junctions (zonulae adherentes) between photoreceptor cells and Müller cells; this layer provides structural integrity.
4. Outer Nuclear (Granular) Layer: The layer that harbors the nuclei of the photoreceptor cells.
5. Outer Plexiform (Synaptic) Layer: The site where photoreceptor cells synapse with bipolar cells.
6. Inner Nuclear (Granular) Layer: This contains the nuclei of bipolar cells, horizontal cells, and amacrine cells.
7. Inner Plexiform (Synaptic) Layer: This layer is a region that facilitates the synaptic connections between bipolar, amacrine, and ganglion cells.
8. Ganglion Cell Layer: Comprising the ganglion cells, this layer serves as the output structure for transmitting visual information to the brain.
9. Optic Nerve Fiber Layer: Constituted by axons emanating from the ganglion cells, this layer contributes to the formation of the optic nerve.
10. Inner Limiting Membrane: This layer serves to demarcate the retina from the vitreous humor, acting as a boundary.

Upon entering the eye, a light ray embarks on a journey that commences at the cornea, traverses the aqueous humor, progresses through the lens, and navigates through the vitreous humor before culminating at the retina [38]. Within the retinal structure, diffused light is absorbed by the pigmented epithelium, while direct light impinges upon photoreceptor cells, namely cones and rods [39,40]. A crucial consideration in this process is the intricate relationship between photoreceptor cells and the pigmented epithelium. This is embodied in the phenomenon known as the retinal visual cycle. This cycle encompasses the re-isomerization of *trans*-retinol, the inactive component, to cis-retinol, the active component, a conversion facilitated by pigmented epithelial cells [41,42]. In photoreceptor cells, retinol covalently binds to opsin, forming 11-*cis*-retinal, the active isomer. Upon the absorption of photons from visible light, these photoreceptor cells convert *cis*-retinal into 11-*trans*-retinal. This molecular transformation induces a conformational change in the protein, thereby initiating the visual process. In essence, these photopigments detect and transduce light stimuli into electrical signals through a cascade of biochemical events termed phototransduction. Given that the *cis*-retinal isomer is requisite for maintaining photoreceptor activation, it must be reconverted to its original *cis* form. This reconversion occurs exclusively in the pigmented epithelium through a series of enzymatic reactions executed within these cells [43,44].

Wavelengths themselves do not possess inherent color; however, they are commonly grouped into categories based on perceived coloration: short wavelengths are associated with the color “blue”, medium wavelengths correspond to “green”, and long wavelengths are identified as “red” [45]. For colors to be perceived, photoreceptor cells, such as cones, play a pivotal role in photopic vision, requiring bright light conditions. Cones are specifically endowed with photosensitive pigments known as opsins. Each subtype of cone contains a unique pigment, conferring sensitivity to a specific range of wavelengths. Erythropsin in type L cones is most sensitive to long wavelengths around 700 nm, where red light is prevalent. Chloropsin in type M cones responds to medium wavelengths situated between 530–570 nm, corresponding to green light. Cyanopsin in type S cones shows the greatest sensitivity to short wavelengths around 430 nm, where blue light is localized [46,47].

Notably, cone responses do not directly inform us about the absorbed wavelengths but rather the total number of absorbed photons, a principle known as univariance [48]. On the other hand, rods are responsible for scotopic vision, which operates under low-light conditions. These photoreceptors contain the photosensitive pigment rhodopsin, a protein highly sensitive to wavelengths near 500 nm, where green–blue light is concentrated. Remarkably, rods can detect a single photon of light [49,50,51]. Thus, the specialized photoreceptors—cones for photopic vision and rods for scotopic vision—enable the diverse range of human visual experiences. Each type contributes uniquely to the perception of color and luminance, shaped by their respective spectral sensitivities.

Upon the absorption of light, photoreceptor cells undergo a chemical change that is subsequently converted into an electrical signal. This electrical signal is transmitted to bipolar cells [52], with which they make synaptic contacts. Further, the signal proceeds to ganglion cells, where the optic nerve is formed. This nerve serves as the conduit for transmitting visual information to the brain for processing and interpretation [53,54]. The optic nerve, formed by axonal extensions of ganglion cells, is sensory in nature and connects the neural retina to the brain. These nerves are unmyelinated within the retina but become myelinated, as other central nervous system axons, by oligodendrocytes upon exiting it [55]. Structurally, the optic nerves are cylindrical and paired, comprising approximately 770,000 to 1.7 million fibers originating from ganglion cells [56]. The optic nerve head is primarily composed of four regions: the nerve fiber layer, the prelaminar region, the lamina cribrosa, and the retrolaminar region. Upon traversing the lamina cribrosa, the nerves are coated with pia mater and dura mater and proceed through the optic canal of the sphenoid bone, above the diaphragm sellae and into the suprasellar venous sinus [57].

Beyond the ocular globe, the optic nerve is categorized into three segments: intracranial; intracanalicular, located within the optic canal and the lesser wing of the sphenoid bone; and pre-chiasmal, before the optic chiasm formation [58]. At the optic chiasm, fibers from each eye intersect such that the optic tract comprises both ipsilateral temporal fibers and contralateral nasal fibers. Most of these fibers synapse in the lateral geniculate body of the thalamus and terminate in the visual cortex, also known as the striate cortex or V1 or Brodmann Area 17 (BA17). A minority of these fibers project into the pretectal nucleus of the midbrain via tectospinal or tectobulbar pathways, synapsing in motor centers for the control of pupillary reflex relative to light intensity. Others project to the superior colliculus, also in the midbrain, which regulates saccadic eye movements. Fibers projecting to the suprachiasmatic nucleus in the hypothalamus are involved in perceiving diurnal light changes and sending information to the pineal gland.

In general terms, visual information is conveyed from the eyes to the retina and subsequently converges in the lateral geniculate nucleus (LGN) of the thalamus. From the LGN, signals are routed ipsilaterally to their destination, the visual cortex located in the occipital lobe. Within the visual cortex, stimuli are processed and integrated to make sense of incoming information, resulting in the perception of distance, depth, motion, shape, and color. This integrative process occurs within a neural network distributed across various areas and pathways in the visual cortex. These areas include the primary or striate visual cortex (V1) and extrastriate cortical regions (V2, V3, V4, V5, V6). Each of these regions corresponds to Brodmann areas; specifically, V1 corresponds to Brodmann Area 17, while V2 through V5 are situated within Brodmann Areas 18 and 19. Functional specialization exists within these cortical regions, each responding to specific types of visual stimuli. For example, V2 receives direct input from V1 and is primarily involved in color perception and orientation. V3, which receives projections from both V1 and V2, is associated with shape perception. Region V4 is predominantly engaged in color processing, and V5 is crucial for motion analysis [59,60,61].

### Optic Neuritis

Diseases of this cranial nerve include the optic neuritis, an inflammatory disorder of the optic nerve that predominantly affects women and individuals between the ages of 20 and 50. The condition is marked by a gradual loss of vision, typically unilateral, progressing over hours to days. Symptoms commonly encompass ocular pain exacerbated by eye movement, central visual impairment, diminished visual acuity, altered color perception, and an abnormal pupillary response. The extent of visual loss may vary depending on the location of the lesions within the optic pathway. Thus, lesions situated posterior to the optic chiasm can lead to distinct visual impairment patterns compared to those located anterior to the chiasm. For instance, anterior lesions can result in monocular visual defects, while posterior lesions can affect the visual field in both eyes due to the crossing of optic nerve fibers at the chiasm. Optic neuritis is often associated with demyelinating diseases, such as multiple sclerosis (MS), where the pathogenesis typically involves an autoimmune response, leading to inflammation and demyelination of the optic nerve [62,63].

## 4. Cranial Nerve III: Oculomotor Nerve

The third cranial nerve, also known as the oculomotor nerve, plays a pivotal role in regulating eye movements. This nerve not only innervates the muscles responsible for eye motion but also includes a parasympathetic component essential for controlling pupillary and extraocular muscle functions. It originates from a nucleus located in proximity to the midline of the mesencephalon, specifically at the level of the superior colliculus [64]. The neurons of this nerve are primarily found in two distinct regions: the general somatic efferent component (GSE) and the parasympathetic component. The nerve terminals of the GSE neurons innervate the extrinsic eye muscles and traverse anteriorly to the red nucleus before exiting from the anterior surface of the mesencephalon through the interpeduncular fossa [65,66]. The GSE component is responsible for voluntary contractions of the extrinsic eye muscles that govern eye movements within the ocular orbit. Additionally, it controls the levator palpebrae superioris muscle, responsible for eyelid elevation [67].

The parasympathetic component of the nerve originates from the Edinger–Westphal nucleus, which is situated within the mesencephalon [68,69]. Recent research has delineated that this nucleus is part of the broader oculomotor nuclear complex, consisting of two cellular populations: the preganglionic Edinger–Westphal (EWpg) and central projection Edinger–Westphal (EWcp) populations. The EWpg population is considered a part of the oculomotor nuclear complex and is responsible for sending parasympathetic fibers ipsilaterally to innervate the medial and inferior rectus muscles [70,71].

The complex pathway of the oculomotor nerve involves traversing specific arteries and entering specialized anatomical sites, contributing to its multifaceted functionality. The nerve passes between two critical arteries: the superior cerebellar artery and the posterior cerebral artery. Subsequently, it enters the sphenoid bone at a location referred to as the cavernous sinus. Upon exiting this region, the somatic component of the nerve divides into an upper division, which innervates the superior rectus muscle and the levator palpebrae superioris muscle, and a lower division [72,73]. The parasympathetic component continues its course in conjunction with the lower division, exiting the skull via the superior orbital fissure. This allows it to enter the orbit where it innervates the inferior oblique, medial rectus, and inferior rectus muscles. At this point, the sympathetic nerve branches off, giving rise to the ciliary branch [74].

The ciliary branch enters the ciliary ganglion, where it synapses with a cluster of motor neurons. The terminals of these neurons innervate the ciliary muscle and the pupillary constrictor muscle, both located within the eyeball. The primary function of the pupillary constrictor muscle is to contract the pupil, maintaining its normal physiological state, which is in contrast to sympathetic innervation that promotes pupillary dilation. Another muscle controlled by the ciliary branch is the ciliary muscle, encircling the lens’s circumference. When the ciliary muscle relaxes, the lens assumes a flattened shape referred to as an unaccommodated lens. This state is observed when focusing on distant objects, as the lens remains unaccommodated [75]. Conversely, for near vision, the ciliary muscle contracts, causing the lens to become more convex. This alteration in lens shape enhances the clarity of nearby objects by focusing the image directly onto the retina.

### Oculomotor Nerve Disfunctions

The entry of light triggers reflexes at the Edinger-Westphal nucleus, which originates from the pretectal olivary area in mammals. Neurons in the pretectal nuclei respond to varying light intensities and play a crucial role in mediating unconscious behavioral responses to acute changes in light. Light stimulation of this area induces pupillary constriction, while lesions result in a loss of the pupillary reflex [76,77]. When an object appears in the visual field, not only are the eyes oriented towards it, but often head and body movement are required for accurate object localization. These movements occur in a highly coordinated manner, facilitating a precise object location. The complete dysfunction of the oculomotor nerve can lead to exotropia, or outward eye deviation, and eye elevation, resulting in diplopia (double vision). Additional symptoms may include ptosis, the inability to lift the eyelid.

## 5. Cranial Nerve IV: Trochlear Nerve

The fourth cranial nerve, also known as the trochlear nerve, plays a vital role in regulating ocular muscles, contributing significantly to binocular vision coordination and the ability to track moving objects. Its fibers originate from a unique source, the somatic efferent column of cells (SECC). Questions have arisen regarding the SECC as the nucleus’s origin due to its ventral midbrain location. Seminal work by Santiago Ramón y Cajal in 1911 provided empirical evidence supporting its distinction from other columnar neurons. The pathway of these fibers has been a subject of debate, with some researchers suggesting crossing to the opposite side [78]. The trochlear nerve was initially omitted from Galen’s list of cranial nerves, likely due to its accidental removal during brain extraction procedures. It was not until 1664 that Thomas Willis expanded the list, accurately describing the fourth nerve and labeling it the “pathetic” nerve due to its role in governing eye movements in response to emotional and natural instincts [79].

The trochlear nerve is thin and elongated, making it susceptible to injury. It is the thinnest among cranial nerves in terms of the axonal count [80]. During embryological development, it originates from the mesencephalon and crosses within the brainstem. After emerging from the brainstem, it follows the lateral wall of the cavernous sinus, entering through the superior orbital fissure to supply the contralateral superior oblique muscle. The superior oblique muscle’s primary functions include the intorsion, depression (adduction focusing), and abduction of the eye [81]. As the nerve courses downward around the cerebral peduncles before branching towards the cavernous sinus and orbit, it runs alongside cranial nerve III, between the posterior cerebral and superior cerebellar arteries [82,83,84]. It then innervates the superior oblique muscle. This muscle originates near the common tendinous ring on the orbital roof, and its tendon passes through the trochlea, a fibrous loop in the frontal bone. It inserts at the posterior half of the eye.

The trochlear nucleus, located in the midbrain near the midline, innervates primarily the contralateral superior oblique muscle. The crossed axons emerge from the dorsal midbrain, caudal to the inferior colliculus, to form the fourth cranial nerve. It courses ventrally around the cerebral peduncle and continues in the interdural segment, along with its dural sheath, running along the cavernous sinus’s lateral wall. Due to its diminutive size, high-resolution T2-weighted magnetic resonance images are required for visualization.

### 5.1. Congenital Cranial Denervation

A group of diseases known as congenital cranial denervation disorders results from the atypical growth or axonal connections in cranial nerve nuclei, leading to abnormal innervation patterns affecting facial and ocular musculature. High-resolution, thin-section MRI aids in diagnosis, including congenital oculomotor nerve paralysis, congenital trochlear nerve paralysis, Möbius syndrome, Duane retraction syndrome, congenital extraocular muscle fibrosis, synergistic divergence, and synergistic convergence [85].

### 5.2. Superior Oblique Miokymia

An uncommon disorder affecting the fourth cranial nerve, superior oblique myokymia is characterized by sudden, monocular episodes of high-frequency, low-amplitude eye movements. These can occur both vertically and torsionally. Patients often report monocular oscillopsia, characterized by images that appear to move upward and occasionally experience vertical diplopia. Episodes may last from a few seconds to several minutes and can recur multiple times per day, gradually tapering off over weeks, months, or even years. Although triggered by looking in the direction of the superior oblique muscle, most cases do not have any identifiable precipitating factors. Diagnosis primarily entails a comprehensive medical history, as eye movements are subtle and best observed through slit-lamp examination during clinical evaluation [86].

### 5.3. Brown’s Syndrome

Brown’s syndrome, first documented in 1950, manifests as a restriction in upward eye movement during adduction, often accompanied by positive forced ductions in the same gaze position. While generally congenital, the syndrome can also arise from external factors, such as inflammation, trauma, or surgeries affecting the superior oblique tendon or trochlea. The origin of Brown’s syndrome can be either structural or innervational. When the cause is related to innervation, it falls under the broader category of congenital cranial denervation disorders. Affected individuals typically adopt an atypical head posture, characterized by an upward tilt and elevated chin. This unique head position helps to avoid diplopia and facilitate binocular vision; thus, amblyopia is uncommon. The recommended treatment varies with the severity of the ocular misalignment. For milder cases, observation may suffice. Surgical interventions can include superior oblique tenotomy or recession, supplemented with silicone expander placement in the superior oblique muscle. For inflammation-induced Brown’s syndrome, corticosteroids, administered through local or systemic injections, and nonsteroidal anti-inflammatory drugs have been employed.

### 5.4. Ocular Neuromyotonia

Ocular neuromyotonia is a rare disorder affecting ocular motility, characterized by involuntary contractions of one or more extraocular muscles. These spasms may occur spontaneously or may be triggered by focusing on a particular point for an extended period. The underlying etiology is hypothesized to be due to irregular and recurrent discharge of one of the cranial nerves responsible for controlling eye movements, leading to persistent and inappropriate contraction of the extraocular muscles. In instances where ocular neuromyotonia affects the superior oblique muscle, the patient will exhibit hypotropia of the affected eye, often accompanied by exotropia, and will be unable to elevate the eye during adduction. Therapeutic interventions that have shown efficacy in treating ocular neuromyotonia include membrane-stabilizing medications, such as gabapentin, carbamazepine, lacosamide, and phenytoin.

## 6. Cranial Nerve V: Trigeminal Nerve

The intricate framework responsible for processing sensory information and executing motor responses relies on the interaction between the central and peripheral nervous systems. Within this complex system resides the fifth cranial nerve pair, commonly known as the trigeminal nerve. This cranial nerve plays a crucial role in transmitting both sensory and motor signals to and from facial structures, contributing significantly to various daily functions, including the transmission of painful stimuli, facial tactile perception, and mastication. As our understanding of the anatomy, physiology, and associated disorders of the trigeminal nerve advances, its profound impact on an individual’s quality of life becomes increasingly evident. Despite this, clinical management remains a significant challenge.

The trigeminal nerve carries sensory innervation and somatic supply to the face through three main branches: ophthalmic or V1, maxillary or V2, and mandibular or V3. The V3 branch provides motor innervation to masticatory muscles, including the mylohyoid, the anterior belly of the digastric, and the tensor veli palatini and tensor tympani muscles. While this cranial nerve lacks autonomic fibers, some of its branches combine with parasympathetic fibers from other cranial nerves to innervate glands such as the lacrimal, parotid, submandibular, and sublingual glands [87,88,89].

Classified as a mixed nerve, the trigeminal nerve comprises fibers that provide general sensory innervation to head structures, except for the occipital and retroauricular regions, which receive innervation from branches of the cervical plexus and the facial nerve or Cranial Nerve VII. The trigeminal sensory nuclei in the brainstem serve as endpoints for sensory fibers responsible for pain, temperature, touch, pressure, vibration, and proprioception in the skin of one side of the face and head, extending from the vertex forward. These fibers also innervate the conjunctiva, eyeball, part of the external tympanic membrane, mucosa of the anterior two-thirds of the tongue, palate, nasal cavities, paranasal sinuses, meninges above the tentorium (supratentorial), and upper and lower dental arches [90]. Motor fibers, known as special visceral or branchial efferents, originate from the trigeminal motor nucleus to innervate muscles derived from the first pharyngeal or branchial arch.

The spinal tract nucleus of the trigeminal nerve comprises three distinct segments: pars oralis, pars caudalis, and pars interpolaris. These subnuclei transmit sensations of pain and temperature. The primary sensory nucleus processes tactile sensations and light touch. The mesencephalic nucleus handles the transmission of facial proprioception. All these nuclei communicate with the ventral posteromedial nucleus of the thalamus, where they synapse before proceeding to the primary somatosensory nucleus located in the parietal lobe. The motor nuclei supply innervation to the muscles of the first branchial arch, including the masticatory muscles [91].

The ophthalmic division, also known as V1, represents the smallest of the three divisions of the trigeminal nerve and is responsible solely for sensory or afferent functions. It supplies sensory branches to various areas, including the ciliary body, cornea, iris, lacrimal gland, conjunctiva, nasal cavity mucous membrane, frontal sinus, sphenoidal sinus, forehead skin, nose, eyebrows, eyelids, dura mater, and the posterior region of the falx cerebri. Additionally, it provides sensitivity to the skin and mucous membranes of the nasal bridge, medial half of the upper eyelid, medial forehead region, and scalp up to the vertex. The lacrimal nerve conveys information from the lateral upper eyelid [92].

In contrast, the intermediate-sized maxillary nerve, also known as the superior maxillary nerve, represents the second division of the trigeminal nerve. It provides sensory innervation to structures in and around the maxillary bone and the middle area of the face, including the skin of the mid-face, lower eyelid, lateral nose region, upper lip, nasopharynx mucous membrane, palate, maxillary sinus, soft palate, palatine tonsils, gums, and maxillary teeth. The maxillary nerve comprises four groups of branches based on their anatomical origin: those from the skull, the pterygopalatine fossa, the infraorbital canal, and the face. It extends anteriorly over the dural lateral wall of the cavernous sinus, positioned beneath the ophthalmic nerve. It enters the middle cranial fossa through the foramen rotundum, supplying innervation to the supratentorial meninges of the anterior and middle cranial fossae. Additionally, it provides sensitivity to the skin and mucous membranes between the lower eyelid, nasal cavity, and palate. The maxillary nerve forms from the convergence of four distinct nerve branches: zygomatic, infraorbital, superior alveolar, and palatine.

The mandibular division of the trigeminal nerve, the largest among its three divisions, is also known as the inferior maxillary nerve or nerve mandibularis. Unlike the purely sensory ophthalmic and maxillary divisions, the mandibular division is a mixed nerve, encompassing both sensory and motor functions within its various branches. This division consists of two distinct roots: (1) a significant sensory root originating from the lower angle of the Gasserian ganglion and (2) a smaller secondary motor root originating from the pons of the central nervous system. It carries sensory innervation from the teeth, gums, skin of the temporal area, lower third of the face, lower lip, and ear as well as motor innervation to the muscles of the first branchial arch, including the muscles of mastication, tensor veli palatini, tensor tympani, mylohyoid muscle, and anterior belly of the digastric muscle. In the infratemporal fossa, the mandibular nerve gives rise to four main branches associated with the buccal, auriculotemporal, inferior alveolar, and lingual nerves.

### Pathological Conditions and Lesions

In pathology, trigeminal neuralgia (TN) is the most prevalent type of craniofacial neuropathic pain, known for producing excruciating pain. It affects an estimated 4 to 13 individuals per 100,000 per year. The pain is characterized by sharp, sudden sensations resembling electric shocks or burning, typically localized to regions innervated by one or more branches of the trigeminal nerve. TN has three types: idiopathic trigeminal neuralgia (without identifiable causes), classical trigeminal neuralgia (associated with neurovascular compression at the trigeminal root entry region), and secondary trigeminal neuralgia (caused by underlying medical conditions, including multiple sclerosis) [93]. Carbamazepine remains the preferred treatment, with second-line medications, such as lamotrigine and baclofen. In some cases, gabapentin in conjunction with local ropivacaine injections may be effective.

When the motor root of the trigeminal nerve is affected by isolated lesions, it can lead to atrophy and weakness of the masticatory muscles, resulting in jaw deviation towards the opposite side and flaccidity of the mylohyoid and anterior belly of the digastric muscles. Rarely, the abnormal or excessive activity of the masticatory muscles can occur in motor root lesions, leading to hemimasticatory spasm, characterized by involuntary, unilateral, and paroxysmal contractions of the temporalis and masseter muscles.

Pathological processes affecting the trigeminal nerve may also result from extracranial factors, including head and neck neoplasms, metastatic tumors, and inflammatory conditions, such as orbital pseudotumors, abscesses, and sinusitis. Neurovascular compressions affecting the trigeminal nerve in the prepontine cistern, particularly by the superior cerebellar artery, can lead to treatment-resistant trigeminal neuralgia [94].

The optimal management of pathological processes affecting the trigeminal nerve involves a precise examination of the affected anatomical section of the nerve. Sensory disturbances and symptoms may vary, including pain, burning sensations, numbness, or a loss of sensitivity. Specific clinical symptoms depend on the division of the trigeminal nerve involved. When the V1 (ophthalmic), V2 (maxillary), or V3 (mandibular) divisions are compromised, patients may experience various sensory or motor issues. Examination and diagnosis should consider the affected nerve division to tailor appropriate treatment [95].

Trigeminal nerve lesions can range from minor hematomas to complete nerve rupture, often resulting from physical trauma, such as dental extractions. The extent of nerve alteration is categorized by Seddon’s classification, comprising three types: neuropraxia, axonotmesis, and neurotmesis [96].

## 7. Cranial Nerve VI: Abducens Nerve

The abducens nerve, or Cranial Nerve VI, plays a crucial role in nervous system. Specifically, it regulates a vital extra-ocular muscle, the lateral rectus, which is essential for eye movement. The nerve’s name is derived from its unique function in innervating the lateral rectus muscle, responsible for abducting or moving the eyeball outward. This motor nerve is pivotal in coordinating eye movements, is pivotal for ensuring visual stability, and is essential for focus and spatial perception. Anatomically, the abducens nerve consists solely of motor fibers originating from a single nucleus located in the pons, proximal to the facial nerve nucleus. It is characterized by a medial eminence protruding from the floor of the fourth ventricle. Although some species, such as chickens, rabbits, and humans, were once believed to possess an accessory nucleus, it is now understood that this accessory nucleus is absent in primates. In species where it exists, it often associates with Cranial Nerves V and VII, contributing to ocular protection.

The nerve emerges from the brainstem, courses toward the cavernous sinus, and enters the superior orbital fissure before reaching the orbital cavity. It is particularly susceptible to injury among extraocular muscles due to its sharp turn at the petrous portion of the temporal bone before entering the cavernous sinus. Notably, it is the only cranial nerve to innervate an extraocular muscle typically affected bilaterally. Damage to this nerve results in uncoordinated horizontal eye movements and an inability of the affected eye to abduct laterally.

The axons of this nerve originate from the ventral aspect of the brainstem at the junction between the pons and the medulla. These axons travel rostrally and somewhat laterally within the subarachnoid space of the posterior cranial fossa before penetrating the dura mater lateral to the dorsum of the sphenoid’s sella turcica [97]. These axons originate from lower motoneurons in the nucleus and interneurons that transmit signals via the medial longitudinal fasciculus to the lower motoneurons of the opposite oculomotor nucleus’s medial rectus muscle. This intricate neuronal network ensures the smooth coordination of lateral eye movements. Damage to these axons leads to medial strabismus and the loss of the nictitating reflex, which is typically used to assess the nerve’s normal function [98].

Embryologically, this cranial nerve originates from the basal plate of the embryonic pons. As it ascends from the ventral pontomedullary junction, it encounters arteries and veins of the posterior circulation, including the anteroinferior cerebellar artery. Remarkably, the interdural segment of this nerve is notably longer than that of other oculomotor cranial nerves. It enters the petroclival venous confluence, passes through Dorello’s canal, and eventually enters the cavernous sinus, adjacent to the cavernous segment of the internal carotid arteries. The foraminal segment of cranial nerves VI, III, and IV passes through the superior orbital fissure to enter the extraforaminal segment of the orbit, where it innervates the lateral rectus muscle between its posterior and anterior two-thirds [99].

### 7.1. Abducens Nerve Dysfunctions

Dysfunction of the abducens nerve can result from central lesions affecting its nucleus, such as strokes, particularly in the pons, or space-occupying lesions, such as aneurysms, abscesses, or tumors. Elevated intracranial pressure due to subdural hematomas, sagittal sinus thrombosis, or abscesses can compromise the nucleus or efferent fibers of the abducens nerve as they diverge from the brainstem toward the orbit, leading to nerve malfunction. Cerebrospinal fluid (CSF) leakage has also been associated with traction on the abducens nerve, potentially causing sixth nerve palsy [100]. Determining if sixth nerve palsy exclusively affects this nerve is challenging, as it often coincides with dysfunction in other neurological structures. The causes of unilateral abducens nerve palsy may include trauma, vascular deficiencies (often related to diabetes), lumbar punctures (which can result in slight brain sagging due to CSF loss), stretching (as in a pinched nerve), tumors, and multiple sclerosis (MS). The likelihood of recovery ranges from 30% to 60%, with lower rates in cases of bilateral palsy. The majority of abducens nerve disorders manifest as lateral rectus muscle palsy. Clinically, this is often observed as the patient’s inability to focus on an object located on the side affected by the disorder. Unilateral and acquired paralysis of the sixth cranial nerve is the most common clinical presentation.

### 7.2. Microvascular Ischemia

Microvascular ischemia is the most frequent etiological factor, and individuals with preexisting conditions, such as diabetes, hypertension, or atherosclerosis, are more susceptible to developing sixth nerve palsy. While rare, congenital sixth nerve palsy can occasionally be associated with complications during childbirth or other intrinsic disorders [100]. Patients with microvascular ischemia often experience a sudden onset of diplopia (double vision), often accompanied by pain. To alleviate symptomatic double vision, these patients may use an eye patch on the affected eye.

### 7.3. Diplopia and Petrositis

The diplopia is typically horizontal and binocular, worsening when the patient looks toward the affected side. Additionally, the patient may tilt their head toward the affected eye to mitigate the effects of double vision. Other neurological symptoms, such as headaches, nausea, and vomiting, may also be present and should prompt further diagnostic evaluation. Infections of the petrous temporal bone, known as petrositis, can compromise the nerve, resulting in lateral rectus weakening and the medial deviation of the affected eye, a condition referred to as Gradenigo’s syndrome [100].

## 8. Cranial Nerve VII: Facial Nerve

The seventh cranial nerve, also known as the facial nerve, is a composite entity encompassing motor, general sensory, special sensory, and visceral components. It originates embryologically from the second pharyngeal arch and emerges ventrolaterally from the lower pons. Significant milestones in the understanding of this nerve include Gabriel Fallopius’ seminal work in 1550 detailing its course through the temporal bone. In 1829, British neurologist Sir Charles Bell made another groundbreaking contribution by distinguishing that, while cranial nerve V handles sensory innervation of the face, cranial nerve VII controls its motor function [101,102,103]. We now know that the facial nerve is responsible for various functions in the head and neck. It innervates the muscles responsible for facial expression, motor fibers of the middle ear, taste receptors in the anterior two-thirds of the tongue, parasympathetic fibers to the salivary glands, and somatic afferent fibers to the external auditory canal and auricle [104,105].

The complex trajectory of the facial nerve begins from its origin in the pons and extends to its terminal distribution in the face. The motor nuclei of this nerve are located beneath the floor of the fourth ventricle. As the nerve extends, its motor fibers encircle the nuclei of the abducens nerve (CN VI). Upon exiting the pons at the cerebellopontine angle, these motor fibers take an anterolateral direction. While traversing the pons, these motor fibers intersect with special visceral sensory fibers originating from the solitary nucleus, responsible for taste sensation. They also intersect with parasympathetic motor fibers that regulate salivary gland function [106]. The nerve enters the temporal bone through the internal auditory meatus. Within the petrous part of the temporal bone, it navigates through the facial canal, which is divided into three segments: labyrinthine, tympanic, and mastoid. Originating from the base of the skull, the nerve exits through the stylomastoid foramen and courses through the parotid gland. The mastoid segment gives rise to the nerve of the stapedius muscle and the chorda tympani. The sensory fibers originating from receptors in the anterior two-thirds of the tongue reach the geniculate ganglion via the chorda tympani. These fibers traverse along the nervus intermedius through the internal auditory meatus and the cerebellopontine cistern, ultimately terminating in the solitary tract nucleus. The parasympathetic fibers of the nervus intermedius originate from the superior salivary nucleus. At the geniculate ganglion, the greater petrosal nerve supplies parasympathetic fibers to the lacrimal gland as well as to the mucosa of the mouth, nose, and pharynx [107,108,109].

In humans, this mixed nerve comprises approximately 7000 fibers, the majority of which are myelinated and range in diameter from 7 to 10 μm. The superior terminal motor branches of the facial nerve traverse the parotid plexus and advance anteriorly through the zygomatic arch towards the frontal, orbicular, and corrugator muscles. Additional branches extend horizontally towards the zygomatic, orbicularis oculi, and adjacent muscles enveloping the buccal region, including the buccinator and masseter muscles. Furthermore, an inferior cervical branch innervates the platysma muscle, which lies superficially and contributes to skin tautness in the neck. This muscle may become visibly prominent during facial gestures. According to traditional anatomical teachings, there are five recognized terminal branches of the facial nerve: the temporal, zygomatic, buccal, marginal mandibular, and cervical branches. 

The motor function that supplies the muscles responsible for facial expressions as well as the stapedius muscle in the ear originates from the facial nucleus within the pons. This motor component then courses alongside cranial nerve VI [110]. Predominantly, the study of the seventh cranial nerve focuses on its somatic motor component, which is responsible for innervating muscles present in the face and, to a lesser extent, the neck. In total, this nerve innervates 28 muscles, each with various roles in controlling the movement of the eyelids, eyebrows, lips, mouth, cheeks, and chin [111].

### 8.1. Pathophysiological Conditions

The facial nerve is implicated in various diseases, the most common of which is Bell’s palsy. This condition is characterized by facial paralysis, and its etiology often remains idiopathic. Several theories suggest that vascular spasms of the arteries in the facial canal, which supply blood to the nerve, may contribute to this condition. Alternatively, the inflammation and swelling of the nerve within the bony canal may also play a role in the development of Bell’s palsy. Facial paralysis has substantial impacts on both functionality and appearance, resulting in profound psychological and aesthetic challenges. Numerous approaches for relief have been explored, including primary repair and the utilization of nerve grafts or conduits. Tissue engineering has emerged as a pivotal field in the development of synthetic materials that can mimic nerve properties, thus minimizing any further patient harm. Moreover, ongoing research is increasingly examining the potential benefits of incorporating neurotrophic factors or stem cells within or around the repair site to further enhance neuronal recovery [112].

Additionally, other conditions can affect the motor functionality of the seventh cranial nerve. These include tumors infiltrating the temporal bone, temporal bone fractures, Ramsay–Hunt syndrome (characterized by herpes zoster affecting the geniculate ganglion and causing severe facial paralysis along with vesicular rashes in the external auditory canal), acoustic neuromas, basilar artery dilatations due to aneurysms, leprosy, and infectious mononucleosis. The latter has the potential to manifest as sudden-onset, single, or multiple cranial paralyses, with bilateral facial paralysis being the most common combination. Furthermore, the motor function of the seventh cranial nerve can also be impacted by myasthenia gravis.

### 8.2. Comparative Features

In other mammals, such as dogs, the facial nerve shares characteristics with its human counterpart. Originating from the rostral and ventral medulla, it controls the muscles responsible for facial expression, taste sensation in the rostral two-thirds of the tongue, and skin sensitivity on the inner surface of the auricle. Typical reactions observed in dogs associated with proper facial nerve function include symmetrical facial expression; normal movements of lips, ears, and eyelids; and the presence of the palpebral reflex. Ears respond to stimulation by moving, and there is an immediate negative taste response. On the sensory level, a behavioral reaction occurs, and the ears contract. Altered mental states or deficits in the seventh cranial nerve also indicate a central vestibular disorder; however, certain polyneuropathies may affect this nerve as well [113].

Rats, serving as a model for rodents, possess a substantial number of facial muscles, amounting to 20 in total, but a debate persists regarding the specific similarities between facial muscles in monotremes and in other mammals [114]. Mammalian facial muscles represent a subgroup of the hyoid muscles, the muscles innervated by the seventh cranial nerve. In strepsirrhines, the facial muscles resemble those in non-primate mammals, such as tree shrews. Notably, there are some differences; strepsirrhines possess a muscle known as the depressor supercilii, which is generally not differentiated in tree shrews. Conversely, strepsirrhines lack two muscles commonly differentiated in these mammals: the zygomatico-orbicularis and the sphincter colli superficialis. In contrast, macaques often lack certain muscles, such as the risorius, anterior auricular, and temporoparietal muscles, which are present in hominids, such as humans. However, they possess muscles that are typically not differentiated in certain hominid groups, for instance, the cervical platysma in orangutans, panins, and humans and the posterior auricular in orangutans.

Lampreys and hagfish are the only representatives of the most ancient branch of vertebrates. The facial nerve in lampreys comprises both motor and sensory fibers and is divided into four primary branches: buccal, hyomandibular, thyroid, and pharyngeal. The buccal branch, located anteriorly, innervates the neuromasts, which are sensory organs situated in the epithelial pit. These neuromasts extend from the front of the eyes to the tip of the snout. It has been documented that this branch fuses with the ophthalmic branch of the trigeminal nerve along its distal course [115]. The efferent nuclei responsible for facial (VII) and octolateral (VIIIeff) functions in adults display significant variability in terms of their spatial arrangement and target destinations. Nonetheless, it has been observed that, in most species, branchiomotor neurons VII (VIIbm) and (VIIIeff) predominantly originate in the r4 region. Consequently, divergent locations found in adult specimens are primarily attributed to variations in the extent of caudal and lateral migration. In lampreys, the VIIbm nucleus can be traced from Mauthner’s cell in caudal r4 to the vicinity just behind the accessory Mauthner cell in caudal r5 across all developmental stages, from early larvae to post-morphic adults. In adult lampreys, the VIIIeff neurons are situated ipsilaterally near both Mauthner’s cell and the VIIbm neurons, suggesting that they derive from the r4 region [116].

## 9. Cranial Nerve VIII: Vestibulocochlear Nerve

The eighth cranial nerve, known as the vestibulocochlear nerve, is responsible for both hearing and balance perception. It comprises two subunits: the vestibular nerve, responsible for detecting head movement and position, and the cochlear nerve, tasked with transmitting auditory signals from the inner ear to the central nervous system. Formerly referred to as the auditory nerve, it exits the brainstem as two subdivisions, with the vestibular segment located ventrally to the cochlear segment [117].

During embryonic development, the otic placode gives rise to both the vestibule and the cochlea. The vestibulocochlear nerve emerges from the pons, travels upward and outward through the cerebellopontine angle, and enters the internal auditory canal (IAC) via the acoustic porus. Within the IAC, it splits into three separate nerve structures: the cochlear nerve, the superior vestibular nerve, and the inferior vestibular nerve. The cochlear nerve lies inferiorly and anteriorly beneath the facial nerve, while the superior vestibular nerve is posterior and superior, and the inferior vestibular nerve is posterior and inferior [118].

Bipolar neurons in the vestibular ganglion, along with efferent neurons, travel in the cochlear and vestibular nerves. The vestibulocochlear nerve has a notably elongated central myelinated section compared to other cranial nerves. This characteristic contributes to a higher incidence of schwannomas originating from this nerve, complicating surgical procedures where differentiation between central and peripheral nerve lesions is crucial. The criteria for this differentiation are the nerve sections proximal to the spiral and vestibular ganglia [119]. The distal axons of bipolar neurons extend to the semicircular canals, innervating the utricle and saccule, entering the cerebellopontine angle, and merging with the vestibular nuclear complex before reaching motor nuclei in the brainstem, upper spinal cord, cerebellum, and thalamus.

The vestibular system includes three semicircular canals, the utricle and the saccule. Maculae, specialized areas within the utricle and saccule, contain ciliated cells acting as sensory transducers. The crests of the semicircular canals also feature these ciliated cells. The membranous labyrinth extends across the saccule and the utricle, each housing a macula covered by a gelatinous layer called the otolithic membrane, composed of small calcium carbonate particles known as otoconia. These otoconia either rest on the otolithic membrane’s surface or are embedded within its upper layer. The primary function of the utricle and saccule is to detect head orientation and movements relative to gravity. When the head moves, gravity pulls the otolithic membrane, causing the ciliated cells’ stereocilia to shift in response.

Sound waves travel through the external auditory canal, vibrating the tympanic membrane. These vibrations pass through the middle ear cavity, aided by the malleus, incus, and stapes (ossicles), reaching the oval window of the cochlea, the spiral part of the bony labyrinth housing the cochlear duct. The ossicles not only transmit sound energy from the tympanic membrane to the oval window but also amplify the pressure applied to the oval window. The cochlea connects to the middle ear cavity via two bony apertures: the vestibular window (oval window), covered by the stapes, and the cochlear window (round window), covered by a thin, flexible diaphragm. As the stapes oscillate within the oval window, pressure waves form in the perilymph. These waves traverse the cochlea, inducing vibrations in the round window’s diaphragm. The round window’s flexibility allows for slight fluid movement, facilitating sound propagation.

The nuclei that constitute the vestibulocochlear nerve are categorized into distinct subgroups:

1. Cochlear nuclei: These small structures in the brainstem play a vital role in auditory processing.
  a. Ventral cochlear nuclei: Located in the anterior and lateral regions of the inferior peduncle.
  b. Posteroventral and anteroventral cochlear nuclei: Encompassed within the anterior part.
  c. Dorsal cochlear nuclei: Situated posteriorly and laterally to the brainstem’s surface, around the cerebellar peduncle.
2. Vestibular nuclei: These neuronal cell bodies reside within the brainstem.
  a. Medial vestibular nucleus: Located in the upper medulla, adjacent to the fourth ventricle’s floor.
  b. Lateral vestibular nucleus: Found in the upper medulla, lateral to the medial nucleus.
  c. Inferior vestibular nucleus: Positioned in the lower medulla, beneath the medial and lateral nuclei.
  d. Superior vestibular nucleus: Located in the upper pons, above the inferior and medial nuclei.

Cochlear nerve damage can result from sensory receptor organ impairment, leading to sensorineural or endocochlear deafness or damage affecting the nerve or central cochlear pathways, known as retrocochlear sensorineural deafness. Various tests, including vocal audiometry, pure-tone audiometry, and auditory evoked potentials, are used to evaluate hearing conditions [120]. Damage to the vestibular nerve can cause symptoms such as vertigo, which affects balance, and nystagmus. When evaluating patients with sudden or severe vertigo, it is crucial to determine if the situation is emergent, confirm the presence of vertigo, localize the anatomical lesion, and assess the potential impact on the individual.

Vertigo, the sensation of motion, can manifest as illusions of environmental movement or self-motion, including tilting, dropping, and lateral pulsion. Oscillopsia, a perceived object oscillation while walking or driving, occurs with open eyes, indicating deficits in vestibulo-ocular reflexes [121]. Benign paroxysmal positional vertigo (BPPV) is a common cause of vertigo characterized by brief episodes of dizziness resulting from otoconia displacement within the semicircular canals.

Dizziness, different from vertigo, is more often associated with medical issues such as cardiovascular and psychiatric conditions than with vestibular disorders. It is essential to differentiate between vertigo and other potential causes, including hyperventilation, presyncope, and imbalance.

Additional vestibular and auditory pathologies include the following:

### 9.1. Vestibular Schwannomas

Vestibular schwannomas, also known as acoustic neuromas, are typically benign tumors that exhibit slow growth in the region of the cochlear-vestibular nerve within the internal auditory canal and/or cerebellopontine angle (CPA). These tumors predominantly arise from Schwann cells of the vestibular nerves and account for approximately 10% of all intracranial neoplasms and 80% of all CPA tumors. Patients presenting with vestibular schwannoma symptoms often exhibit unilateral ontological manifestations, such as asymmetric hearing loss or tinnitus localized to one side of the head. Imbalance is a common symptom, and in certain cases, patients may experience vertigo. Rare instances of facial nerve weakness have been reported. Approximately 10% of cases may result in sudden hearing loss. These tumors are predominantly sporadic and appear exclusively on one side of the head [122].

### 9.2. Meniere’s Disease

The hallmark of Meniere’s disease is the onset of “attacks” characterized by episodes of vertigo, tinnitus, and hearing loss. Vertigo may last from 20 min to several hours, followed by a protracted period of discomfort. Patients are generally asymptomatic between these episodes. Additional symptoms may include postural and gait instability, sudden episodes of falls (known as Tumarkin crises), and nausea. Although the exact incidence of Meniere’s disease remains elusive, estimates suggest 15.3 cases per 100,000 individuals [123].

### 9.3. Vestibular Neuritis

Viral inflammation of the vestibular nerve is believed to result in an imbalance in vestibular tonic activity, leading to vestibular neuritis. If a patient experiences sudden-onset vertigo lasting hours or days, evaluation for vestibular neuritis, labyrinthitis, and other life-threatening causes of acute vestibular syndrome, such as posterior circulation stroke, is imperative. The aim of the medical evaluation, physical examination, and ancillary testing is to confirm the diagnosis of vestibular neuritis and rule out central causes of dizziness, including cerebellar hemorrhage and infarction. Typical symptoms for patients with vestibular neuritis include nausea, vomiting, sweating, and malaise.

### 9.4. Vestibulocochlear Nerve in Canines

In canines, the vestibulocochlear nerve comprises both a vestibular component, responsible for balance, and a cochlear component, responsible for hearing. In instances where an animal has suffered either a central (pertaining to the nuclei and pathways) or peripheral (pertaining to the receptors) vestibular injury, the animal will exhibit symptoms such as vestibular ataxia, nystagmus, a head tilt, and a propensity to lean towards the affected side in the case of a unilateral injury. To determine whether the vestibular syndrome is central or peripheral, a postural reaction assessment should be performed. Hemiparesis is the resultant condition if the lesion is unilateral, while tetraparesis ensues if the lesion is bilateral. When presented with an auditory stimulus, the animal is expected to turn its head toward the sound source. However, the critical factor determining this response is the animal’s level of attentiveness. This process is known to be bilateral.

### 9.5. Congenital Vestibular Disease in Animals

Reports of congenital vestibular diseases have been documented in both feline and canine species. Distinctive breeds of cats, such as the Burmese, Tonkinese, Persian, and Siamese, as well as popular canine breeds, such as Dobermans, fox terriers, German shepherds, and English cocker spaniels, have all been subjects of study. Bilateral congenital vestibular disease has been specifically observed in beagles and dachshunds. Clinical symptoms generally manifest between three and four weeks post-birth. Affected animals may exhibit a range of symptoms, including head tilts, incoordination, spinal curvature, involuntary eye movements, and, in some cases, hearing loss. If the disease affects both hemispheres of the animal’s brain, atypical lateral head movements unrelated to standard nystagmus may occur [124].

### 9.6. Geriatric Vestibular Syndrome in Canines

Canine geriatric vestibular syndrome, also known as idiopathic vestibular syndrome, mirrors its feline counterpart. However, it is common among older dogs and is associated with a sudden-onset peripheral vestibular disease of unknown etiology. These cases are often misdiagnosed as “strokes” due to the abrupt and dramatic presentation of clinical symptoms. Nonetheless, vascular accidents as a cause of vestibular disease are extremely rare in canines. Like in cats, no specific treatment exists for this condition; supportive care, such as controlling vomiting in the initial stages and ensuring adequate nutrition and fluid intake, is recommended. Although cases should resolve spontaneously, some residual symptoms, such as a head tilt, may persist.

### 9.7. Acute Idiopathic Vestibular Syndrome in Felines

Acute idiopathic vestibular syndrome is a prevalent peripheral vestibular disease in cats, characterized by sudden and unexplained onset. Its clinical symptoms are consistent with an abrupt disruption of the peripheral vestibules, including horizontal or rotary nystagmus, a head tilt towards the side of the lesion, leaning or falling to one side, and, in severe cases, vomiting, circling, and tilting towards the side of the lesion. Specific impairments, such as frontal deafness and unilateral deafness, may take time to recover or could be permanent.

## 10. Cranial Nerve IX: Glossopharyngeal Nerve

Cranial nerve IX, also known as the glossopharyngeal nerve, plays a crucial role in connecting the brain with the tongue, pharynx, and certain internal organs. Due to its complex structure and multifaceted functions, this nerve is integral to various activities, such as taste perception, swallowing, blood pressure regulation, and sensation in specific areas of the neck and head. The term “glossopharyngeal” refers to both the tongue and the pharynx. The physical tongue, which weighs between 56 to 85 g in adults, is often larger than commonly perceived. The pharynx, meaning “throat”, is the connecting tube that links the oral and nasal cavities to the esophagus and larynx. The pharyngeal cavities play a role in respiratory and digestive functions as well as in sound production or phonation. Circular constrictor muscles assist in propelling food towards the esophagus during swallowing, while longitudinal muscles elevate the pharynx’s walls above the food bolus.

The glossopharyngeal nerve comprises approximately five to six compact fiber bundles emerging from the medulla oblongata, positioned anteriorly to the vagal fibers. These fiber bundles proceed towards the anteromedial region of the jugular foramen before descending in a curved path, with a convex shape situated posteriorly and inferiorly to the root of the tongue. Here, it bifurcates into its terminal branches. Within the lateral wall of the pharynx, the nerve positions itself and courses medially at the base of the tongue via the lateral area of the stylopharyngeus muscle before entering the tongue. Two sensory ganglia exist within the nerve: the superior ganglion is identifiable as a minor protrusion of the nerve at the jugular foramen, while the larger inferior (petrosal) ganglion resides below it.

According to Wilson-Pauwels [62], the glossopharyngeal nerve encompasses five modalities: general sensory, visceral sensory, special sensory, branchial motor, and visceral motor. The general sensory modality is responsible for providing general sensation to the posterior third of the tongue, tonsils, external ear skin, internal ear surface, tympanic membrane, and pharynx. The visceral sensory modality offers subliminal sensitivity from the carotid body (chemoreceptors) and the carotid sinus (baroreceptors). The special sensory modality, which is unique and distinct, is responsible for transmitting the sense of taste from the posterior part of the tongue. The branchial motor modality innervates the stylopharyngeus muscle. Lastly, the visceral motor modality, specifically the parasympathetic efferent, is accountable for activating the parotid gland and regulating the blood vessels of the carotid body. Furthermore, the nerve plays a variety of other functions, including motor support for the stylopharyngeus muscle, parasympathetic innervation to the parotid gland, and transmitting sensory information from the carotid sinus and carotid body as well as conveying sensory information from the external auditory meatus and the tympanic membrane.

The nuclei associated with the glossopharyngeal nerve include the following [125]:

1. Ambiguous nucleus: Responsible for the motor functions of the stylopharyngeus and superior pharyngeal constrictor muscles, located in the upper part of the medulla oblongata, adjacent to the inferior olivary nucleus, and containing a collection of efferent cell bodies.
2. Inferior salivary nucleus: Serving as a parasympathetic center that activates salivary glands.
3. Inferior solitary nucleus and tract: Responsible for the sensory nucleus that deals with special visceral information.
4. Nucleus of the spinal trigeminal tract: Collecting general somatic information related to the trigeminal nerve.

A study by Doty [126] revealed that the nerve projects to regions previously underestimated, specifically extending to the lateral area of the middle portion of the tongue. This finding corroborates anatomical descriptions from 19th-century textbooks and is substantiated by electrogustometry tests. These implications are both foundational and practical, deepening our understanding of the dorsal tongue’s sensory innervation and providing crucial information for assessing the neural basis of various tongue disorders, including age-related, viral, drug-related, and regional conditions.

### 10.1. Pathological Conditions

Various disorders can affect the glossopharyngeal nerve or its nuclei, such as injuries impacting the ambiguous nucleus; infarcts in the territory of the posterior inferior cerebellar artery; motoneuron disorders, such as amyotrophic lateral sclerosis; or peripheral involvement due to tumors or other lesions in the jugular foramen area.

The rare disorder known as glossopharyngeal neuralgia is characterized by severe, paroxysmal pain localized in the ear, deep in the neck, the tonsil, or the base of the tongue. The pain is triggered by speaking, chewing, and swallowing and can be associated with coughing and, in some cases, syncope due to the afferent activation of the baroreceptors. The etiology of glossopharyngeal neuralgia can be idiopathic but may also be induced by massive lesions, infections, glossopharyngeal neuroma, or eagle syndrome, which is presumed to occur when an elongated styloid process irritates cranial nerve IX [127,128].

In a case study reported by Tomás [129], a considerable tumor in the right cerebellopontine angle was detected. The chief complaint of the 10-year-old patient was hearing loss. Initial imaging led the medical team to suspect a vestibular Schwannoma. However, surgical intervention revealed that the tumor had originated from the glossopharyngeal nerve. Schwannomas affecting this nerve are exceedingly rare and frequently mimic the clinical and radiographic appearances of the more prevalent vestibular Schwannoma.

### 10.2. Comparative Features

In rat snakes, the glossopharyngeal, vagal, and hypoglossal nerves originate from an average of seven small roots located in the posterior part of the myelencephalon. Unlike garter snakes, there is uncertainty regarding whether the three roots arising from the dorsal surface are sensory, and the remaining four roots emerging from the ventral face are motor. These roots typically converge intracranially to form a single craniocervical trunk. A small cluster of sensory cell bodies was found at the root junction through histological examination, representing the combined glossopharyngeal and vagal sensory ganglia. The trunk usually exits the skull through the internal jugular foramen. In some instances, the hypothetical hypoglossal root separates from the other two nerves intracranially, exits the skull through multiple small hypoglossal foramina, and then joins with the extracranial glossopharyngeal and vagal roots [130].

In canines, the glossopharyngeal nerve is responsible for the innervation of the pharyngeal muscles. Furthermore, it gathers sensory information from the internal acoustic meatus and the tympanic membrane, along with the tenth pair of cranial nerves. The nerve also carries gustatory sensations from the root of the tongue and the anterior part of the pharynx. Additionally, the afferent fibers of the nerve terminate in the carotid sinus, responsible for detecting changes in arterial pressure. The parasympathetic component of the nerve innervates the parotid and zygomatic salivary glands.

In lampreys, the glossopharyngeal and vagal nerves are implicated in both sensory and motor functions of the pharyngeal and visceral areas. The glossopharyngeal nerve bifurcates into two main branches: one innervates the epithelial pits located on the dorsal face of the head, whereas the other further divides into two sub-branches. One sub-branch is responsible for activating striated muscles in the first branchial segment and contains respiratory motoneurons for the caudal half of gill 1 and the rostral half of gill 2. The second sub-branch mediates sensory inputs to organs in this region. By 13 days post-fertilization in the larval stage of sea lampreys, the ventral peripheral branch of the glossopharyngeal nerve is observable in P1 prolarvae, emanating from the third pharyngeal pouch. The dorsal branch, on the other hand, appears in P5 prolarvae. Glossopharyngeal motoneurons are solely present in rhombomere 6 (r6) and form a dense nucleus wherein the ventral nerve enters, slightly caudal to the posterior lateral line nerve, near the r6-r7 boundary. Upon entering the brain, two roots are distinguished: a dorsal sensory root composed of thin fibers, and a ventral motor root composed of even finer fibers.

In reptiles such as the *Alligator mississippiensis*, the glossopharyngeal nerve originates in the medulla and is responsible for conveying both sensory and motor signals. It exits the cranial cavity laterally through the jugular foramen located in the occipital bone. Upon exiting, it forms the petrosal ganglion and travels ventrally alongside the external carotid artery. The nerve courses along the dorsal edge of the muscle constrictor colli profundus and between the muscle branchiohyodieus dorsalis and ventralis, supplying innervation to these two muscles. It then passes laterally to the hyoid and medially to the muscle geniohioideus before reaching the ventral edge of the tongue. During ontogeny, the nerve takes an increasingly complex route to its destination in response to the increasingly horizontal orientation of the hindbrain [131].

## 11. Cranial Nerve X: Vagus Nerve

The tenth cranial nerve, commonly known as the vagus nerve, is responsible for approximately 75% of all parasympathetic innervation in visceral organs. It reaches and innervates a multitude of organs, including the respiratory tract, heart, gastrointestinal tract up to the descending colon, pancreas, gallbladder, and liver. The dorsal vagal nucleus is in charge of generating parasympathetic output to the respiratory and gastrointestinal tracts, while the ventrolateral region of the ambiguous nucleus directs output to the heart. Most postganglionic neurons of the vagus nerve are in ganglia forming plexuses near their target tissues [132]. Beyond these roles, the vagus nerve is associated with mood regulation and the stress response, making it crucial in maintaining overall physiological balance or homeostasis.

The sensory nature of the dorsal nucleus of the nerve was once the prevailing belief among prominent anatomists. However, in 1891, Forel introduced the notion that this nucleus was a motor in function and connected to the roots of the vagus nerve. This idea was later substantiated by Marinesco in 1899, who further linked the nucleus to the innervation of smooth muscles in the respiratory and digestive systems rather than the larynx. It was not until the 20th century that the inferior salivary nucleus was assigned to the visceral motor component of the glossopharyngeal nerve. Bechterew, Duval, Obersteiner, and Guden posited that some motor fibers of cranial nerves IX and X originated from a distinct nucleus, situated between the fibers of the hypoglossal nerve and the substantia gelatinosa of the trigeminal nerve, known as the ambiguous nucleus. This nucleus was further subdivided into cell subgroups designated to innervate the muscles of the last pharyngeal arches.

The vagus nerve has an extensive trajectory and impacts multiple bodily functions, such as speech, digestion, respiration, cardiac activity, and possibly the immune system. It traverses the skull, neck, and thorax before terminating near the lower abdominal area in the vicinity of the left colic angle. While this extension is generally acknowledged in textbooks, research suggests that vagal innervation may also extend to pelvic organs, thereby expanding its sphere of influence. The term “vagus” originates from the Latin word meaning “wandering”, aptly describing the nerve’s extensive journey from the brainstem to the large intestine. Emerging from the medulla oblongata as a series of eight to ten fiber rootlets located just dorsal to the inferior olive, the nerve serves as the primary overseer of visceral functions and the regulation of the parasympathetic nervous system. These rootlets converge into a flat cord that exits the skull through the jugular foramen. The nerve houses two separate sensory ganglia, the superior (jugular) and the inferior (nodose). The superior ganglion is situated within the jugular fossa of the petrous temporal bone, which, along with the occipital bone, creates the jugular foramen. In this area, the vagus nerve is near the jugular bulb, an enlargement of the proximal internal jugular vein. The adventitia of the jugular bulb contains the jugular glomus.

The vagus nerve is organized into four groups of fibers, i.e., brachial motor fibers (BMF), parasympathetic fibers (PF), somatosensory fibers (SF), and visceral sensory fibers (VSF). The BMF originate from the ambiguous nucleus in the medulla oblongata; they are primarily responsible for innervating the muscles in the pharynx and larynx and are associated with the third, fourth, and sixth branchial arches. The PF arise from the dorsal motor nucleus, also situated in the medulla oblongata; the preganglionic axons innervate the heart and thoracoabdominal viscera, including the anterior and mid-gut, while the postganglionic neuronal cell bodies are generally located within the walls of their respective target organs, such as the heart and the myenteric plexus. The SF are directed towards the sensory nucleus of the trigeminal nerve; originating from the posterior wall of the external auditory canal and the posterior surface of the tympanic membrane, these fibers proceed through the otic branch of the nerve to the trunk at the jugular foramen, and the cell bodies of these fibers are located in the superior jugular ganglion. The VSF are targeted at the solitary tract nucleus; they encompass taste fibers from the epiglottis and visceral fibers from the hypopharynx, larynx, esophagus, trachea, thoracic, and abdominal viscera, along with pressure and chemoreceptors from the aorta, and their cell bodies are situated in the inferior nodose ganglion.

On the other hand, the nuclei associated with the vagus nerve are the dorsal vagal nucleus (DVN), the ambiguous nucleus (AN), the solitary nucleus (SN), and the trigeminal spinal tract nucleus (TSN). The DVN is located laterally in the fourth ventricle’s floor and contains parasympathetic secretomotor fibers that innervate mucous membranes in the thorax, abdomen, pharynx, and laryngeal mucosa. The AN is in the upper spinal cord, dorsal to the inferior olivary nucleus, housing efferent cell bodies that lead to the striated muscles of the larynx and pharynx, specifically the pharyngeal constrictors. The SN receives gustatory information along with cranial nerves VII and IX, while the inferior solitary nucleus receives visceral sensations via branches towards the nodose ganglion. The TSN receives general somatic information from the ipsilateral side [133].

Specific supply and functions of the vagus nerve are as follows.

### 11.1. Cardiac Innervation

Extensive anastomoses are observed between vagal fibers to the heart and post-ganglionic sympathetic cardiac nerves. These intricate connections facilitate the formation of both ventral and dorsal cardiopulmonary plexuses. These plexuses subsequently give rise to cardiac nerves that are responsible for innervating intrinsic subpericardial ganglia. Notably, the sinoatrial node receives predominant innervation from the right vagus nerve, while the atrioventricular node is principally innervated by the left vagus nerve. Furthermore, the vagus nerve exhibits an inhibitory effect on heart rate, reduces atrioventricular conduction, and prolongs the refractory period of the ventricles.

### 11.2. Respiratory System Innervation

Vagal fibers responsible for signaling the respiratory pathways and lungs traverse via the anterior and posterior pulmonary branches. These fibers significantly contribute to the formation of the pulmonary plexus. The primary physiological outcomes mediated by these fibers include bronchoconstriction and an augmentation in mucus secretion.

### 11.3. Gastrointestinal Innervation

Vagal inputs exert a major influence on the stomach and esophagus but also extend to the proximal small intestine and specific colonic regions. Esophageal branches emerge both above and below the pulmonary branches, culminating in the formation of the esophageal plexus. The anterior and superior portions of the stomach receive innervation from the left vagus nerve, while the posterior and inferior surfaces are innervated by the right vagus nerve. The vagus nerve plays a dual role in gastrointestinal motility: it relaxes smooth muscle in the proximal stomach and stimulates motility in the distal stomach, leading to gastric emptying and increased acid secretion. Within the intestinal tract, vagal efferents exercise modulatory control over various local reflexes that are mediated by neurons of the enteric nervous system. This system comprises sensory neurons, motor neurons, and interneurons that form local integrating circuits and reflexes involved in the regulation of peristalsis and secretion [134].

### 11.4. Vagal Role in Homeostasis

The vagus nerve is also termed the ‘guardian of the body’ because it serves as a complex neuroendocrine–immune network pivotal in maintaining homeostasis. Functioning as a control center, it establishes neuronal connections to various brain regions, assimilating interoceptive information and delivering adaptive modulatory feedback. While most of the central fibers are unmyelinated C fibers originating from visceral organs, myelinated A and B fibers perform critical roles in somatic sensory, motor, and parasympathetic innervation. Primary vagal fibers are cholinergic, yet non-cholinergic and non-adrenergic neurotransmitters are also involved. Recent studies indicate that the vagus nerve also modulates mood, inflammation, and pain [135].

### 11.5. Reflexive Functions

The vagus nerve governs a variety of reflex actions, including swallowing, coughing, yawning, sneezing, and hiccupping. However, only the gag reflex is suitable for needle testing during neurological evaluations. This reflex engages afferent fibers from the glossopharyngeal nerve (and occasionally the vagus nerve) and efferent motor fibers from the vagus nerve to the pharynx, soft palate, and tongue. The reflex is activated by tactile stimulation of the pharynx or the posterior portion of the tongue, resulting in symmetrical contraction and elevation of the pharynx as well as tongue retraction [136]. A patient who exhibits damage to the laryngeal section of the nerve may manifest several symptoms. These include hoarseness, dysphagia, and aspiration when consuming liquids, and the gag reflex becomes impaired. It is noteworthy that the afferent limb of this reflex is carried by cranial nerve IX. In addition, the uvula deviates from the side of the lesion, and the palate fails to elevate [137].

### 11.6. Implications in Eating Disorders

Individuals with bulimia nervosa display an abnormal lack of satiety post-meal, a sensation heavily influenced by the function of the vagus nerve. Additionally, these individuals exhibit higher pain thresholds, which can be modulated by the stimulation of the nerve. Thus, the vagal reflex responsible for satiety and disrupted in bulimic individuals could potentially be stabilized through vagal stimulation, emerging as a potential treatment modality. On the other hand, recent research is exploring vagotomy as a less invasive alternative to gastric bypass surgery for weight loss. This approach suppresses hunger pangs and is occasionally performed in conjunction with gastric banding, leading to an average weight loss of 43% within six months when complemented by a balanced diet and physical activity.

### 11.7. Neurological Syndromes and Vagal Disorders

Various neurological syndromes related to vagus nerve disorders exist, including Wallenberg’s syndrome, Cestan–Chenais syndrome, Vernet’s syndrome, Collet–Sicard syndrome, Garcín’s syndrome, Villaret’s syndrome, and Tapia’s syndrome. These conditions manifest with ipsilateral paralysis and anesthesia of the pharynx and larynx, causing dysphonia and dysphagia [138].

### 11.8. The Vagus Nerve in Dogs

In dogs, the vagus nerve is responsible for innervating the intrinsic muscles located in the larynx, also known as the recurrent laryngeal nerve. It collects sensory information from the mucosa in the larynx and the caudal half of the pharynx. Its parasympathetic component innervates the heart and viscera within the thoracic and abdominal cavities as well as the mucosa in the larynx and pharynx. Clinical indicators include the pharyngeal reflex, which is shared with cranial nerve IX. Lesions on either or both sides, coupled with swallowing difficulties, may result in hoarseness due to cartilage and vocal fold displacement. When stimulated, the animal exhibits auricular movement and head withdrawal, indicating that the vagus nerve serves as an afferent pathway distributed along the seventh cranial nerve.

## 12. Cranial Nerve XI: Accesory Spinal Nerve

Cranial nerve XI, also known as the accessory spinal nerve in honor of Thomas Willis, possesses distinctive anatomical characteristics. Unlike other cranial nerves, the majority of its origin is situated in the spinal cord. Historically, the terms “accessory nerve” and “spinal accessory nerve” were used interchangeably. However, contemporary anatomical literature distinguishes between the two, categorizing the accessory nerve into two distinct components: the spinal root, originating from the spinal cord, and the cranial root, emerging from the brainstem [139]. It ascends through the foramen magnum into the skull before exiting through the jugular foramen. Importantly, this nerve exclusively innervates two muscles, neither of which are in the head, although both are crucial for head movement. These muscles are the sternocleidomastoid and the trapezius, innervated by proprioceptive sensory fibers originating from the primary ventral branches of the second, third, and fourth cervical nerves, which subsequently become part of the extracranial accessory nerve [140]. In the late 19th century, it was discovered that the accessory nerve has two distinct points of origin: one originating from the medullary or caudal part of the medulla oblongata and another from the cranial part. Roller first established the medullary origin in 1881, later confirmed by Van Gehuchten in 1900. Santiago Ramón y Cajal described the morphological features of the nucleus. The cranial origin was located in the caudal portion of the ambiguous nucleus, which provided fibers for the innervation of intrinsic laryngeal muscles.

On the other hand, several authors have proposed that the accessory nerve comprises only the spinal root, separate from the cranial root. An alternative classification system suggests that the cranial root should be designated as the caudal segment of the vagus nerve. This idea has been supported by studies in various animal species. Nevertheless, research on human cadavers has confirmed that the cranial root does indeed play a role in the accessory nerve and constitutes one of the two roots contributing to the accessory nerve in its entirety. Some authors suggest that the cranial fibers diverge from the spinal nerve trunk (referred to as the internal branch) shortly afterward to merge with the proper vagus nerve, situated above the nodose ganglion. These fibers are responsible for innervating the muscles of the larynx and pharynx, including those involved in speech production. Other authors propose that the spinal segment of the accessory nerve progresses toward the subarachnoid spaces located in the anterior portion of the spinal cord before traversing the foramen magnum and joining cranial nerves IX and X along their path through the cistern [141].

A cluster of nerve fibers and nuclei can be found in the lateral section of the ventral gray horn of the cervical spinal cord, where the cell bodies of the accessory spinal motor neurons reside. These appear to be in a caudal extension of the ambiguous nucleus. Other muscles controlled by the ambiguous nucleus are classified as branchiomeric, although there is a debate about whether this applies to the trapezius and the sternocleidomastoid, given their uncertain branchial arch origin. It is worth noting that muscles associated with the branchial arch are linked to cranial extremity and alimentary tube nutrition, whereas the accessory spinal nerve controls head and neck movements during food foraging, as seen in giraffes.

Upon exiting the jugular foramen, the accessory nerve takes a posterior trajectory within the styloid process. It then descends at an angle before finally entering the upper part of the sternocleidomastoid muscle. Some fibers terminate within this muscle, while the remaining fibers traverse it to emerge at the midpoint of its posterior border. These fibers then cross over the posterior triangle of the neck, above the levator scapulae, and are situated very close to the superficial cervical lymph nodes. Approximately five centimeters above the clavicle, the nerve runs beneath the anterior edge of the trapezius muscle to provide its innervation.

Finally, cranial nerve XI is often overlooked in the context of vertebrate neuroanatomy due to its extracranial origin from cervical nerve roots and its trajectory towards cervical musculature. Although it traverses the jugular foramen from the cranial cavity, it is not classified as a cranial nerve. The spinal accessory nucleus has varying locations across different vertebrates. Its motor neurons share structural similarities with those of the hypoglossal nerve. In lampreys, the existence of an accessory nucleus remains unproven. It is posited that lampreys lack a muscle function that functionally correlates with the cucullaris muscle in fish, believed to be homologous to the mammalian trapezius and sternocleidomastoid muscles. Given the absence of a scapular girdle, theoretically, there should be no accessory nerve in these creatures to innervate this musculature. However, recent developments suggest that homologs of the migratory gnathostome muscle precursors from the two rostral somites give rise to the inferior and superior optical muscles in lampreys. In canines, the accessory or spinal nerve comprises two separate roots. The spinal root consists of multiple fibers exiting at the midpoint between the dorsal and ventral roots of the first five spinal nerves. Conversely, the cranial root integrates into the vagus nerve and serves to innervate specific neck muscles, namely the trapezius, omotransversarius, cleidocephalic, and thyrohyoid muscles. Damage to this nerve results in degeneration, weakening, or the complete paralysis of these muscles.

Some clinical manifestations of this cranial nerve are as follows:

### 12.1. Vernet Syndrome

Named after the French neurologist Maurice Vernet (1887–1974), this syndrome is characterized by motor paralysis affecting cranial nerves IX, X, and XI. The most common etiological factors include malignant tumors, aneurysms, or basal skull fractures. Clinical features manifest as severe ipsilateral paralysis and atrophy of the sternocleidomastoid and upper trapezius muscles, coupled with the absence of the gag reflex and diminished sensation on the posterior aspect of the tongue. Primary tumors, such as paraganglioma, meningioma, schwannoma, and metastatic tumors located at the base of the skull, are frequently implicated in Vernet syndrome. However, the syndrome can also result from meningitis, external otitis, sarcoidosis, Guillain–Barré syndrome, and traumatic fractures [142]. Additionally, rare cases have implicated varicella-zoster virus, giant cell arteritis, and acute otitis media as causative agents. Vascular events, such as a large extracranial aneurysm of the internal carotid artery or thrombosis of the internal jugular vein, have also manifested as Vernet syndrome. Iatrogenic cases associated with carotid endarterectomy, leading to edema of the posterior pharyngeal wall or swelling in the parapharyngeal space, have been documented.

### 12.2. Villaret Syndrome

First described by Maurice Villaret (1877–1946), another French neurologist, in 1917, Villaret syndrome was initially discovered in soldiers with multiple injuries to the lower cranial nerves as part of the jugular foramen syndrome. Villaret is known for his research on the precise localization of vascular lesions in the brain. Villaret syndrome occurs when ipsilateral Horner’s syndrome coexists with the impairment of cranial nerves IX, X, XI, and XII. Clinical indications include a loss of taste in the posterior third of the tongue; sensory information loss in the soft palate, dysarthria, ipsilateral tongue deviation, and the triad of ptosis; miosis; and anhidrosis, representing Horner’s syndrome. This syndrome develops from the compression of four lower cranial nerves and fibers of the cervical sympathetic plexus at the base of the skull, particularly in the retroparotid space. Although advanced lung cancer patients frequently exhibit a central nervous system invasion, the presentation of newly diagnosed pulmonary adenocarcinoma with Villaret syndrome is exceedingly rare.

## 13. Cranial Nerve XII: Hypoglossal Nerve

The twelfth cranial nerve, commonly referred to as the hypoglossal nerve, is a purely motor nerve consisting of somatic motor fibers. Its primary function is to control tongue movements, which are essential for tasks such as swallowing, speaking, and breathing. It originates from the hypoglossal nucleus in the medulla oblongata of the brainstem, traverses the posterior cranial fossa, and exits via its dedicated foramen, known as the hypoglossal canal, proceeding to the neck. The nerve comprises a set of fibers that facilitate instinctive tongue movements. Individual differences in specific tongue movements, such as curling, likely result from variations in the neural connections associated with this nerve [143,144].

During embryonic development, the hypoglossal nerve originates from the basal plate of the developing medulla oblongata. It emerges from the posterior and inferior regions of the hypoglossal nucleus within the medulla. The nerve fibers carrying information away from the brain traverse through the medulla in an anterior direction. They exit via the preolivary sulcus located at the anterior and lateral part of the medulla. Subsequently, these fibers extend outward and enter the hypoglossal canal, situated below the jugular foramen and the jugular tubercle in the inferior occipital bone. The nerve continues to the hyoid bone, running between the posterior belly of the digastric muscle, and then along the hyoglossus muscle. Here, the motor branches of the nerve innervate most of the tongue musculature [145,146]. The genioglossus muscle is responsible for protruding the tongue forward from its base, while the hyoglossus muscle functions to retract and depress the side of the tongue. The styloglossus muscle serves to elevate the tongue. The intrinsic tongue muscles, comprising the superior and inferior longitudinal, transversus, and verticalis muscles, play a critical role in altering the tongue’s shape. These muscles enable actions such as shortening, narrowing, and curling the tongue.

Throughout its course, the hypoglossal nerve establishes connections with other nerves at various points. Upon emerging from the hypoglossal canal, the nerve interacts with the inferior ganglion of CN X. At the level of the atlas, it receives sympathetic fibers from the superior cervical ganglion. Lastly, at the base of the tongue, the hypoglossal nerve communicates with the lingual branch of the mandibular nerve, responsible for providing a general tactile sensation to the anterior two-thirds of the tongue [147].

### 13.1. Hypoglossal Nerve Damage

The hypoglossal nerve is susceptible to damage during medical procedures involving the tongue and upper neck region. The clinical consequences of such an injury can vary from tongue atrophy to complex cranial nerve paralysis. Procedures such as head and neck cancer surgeries, anastomosis between the hypoglossal and facial nerves, tumors involving the jugular foramen, and carotid endarterectomy pose a particular risk to the hypoglossal nerve. As a consequence, the implementation of nerve stimulation and monitoring techniques has become indispensable for the accurate identification of the nerve. Such treatment modalities are also employed for managing obstructive sleep apnea in both pediatric and adult populations [148]. Furthermore, injuries to the cranium, meninges, or posterior fossa could result in multiple cranial nerve palsies, including hypoglossal nerve paralysis. It is noteworthy that bilateral hypoglossal lesions are uncommon in the peripheral nervous system, and the simultaneous involvement of both sides of the tongue is typically associated with motor neuron diseases [149].

### 13.2. Unilateral Disfunction

Unilateral hypoglossal dysfunction, characterized by tongue atrophy and weakness, can be attributed to any damage incurred by the hypoglossal nerve. Additionally, progressive unilateral hypoglossal neuropathy could be an outcome of tongue cancer. Notwithstanding, unilateral hypoglossal nerve paralysis is a rare condition affecting various structures, including central, canalicular, neural, or soft tissues. Isolated lesions specifically within the hypoglossal canal are infrequent, with potential causes including schwannomas, synovial cysts, meningiomas, and juxta-articular cysts. In cases where the involvement is below the nuclear level, typical symptoms include tongue deviation towards the side of the lesion, accompanied by unilateral atrophy and lingual fasciculations. The use of imaging techniques is vital for diagnosing hypoglossal nerve lesions, as obtaining a biopsy of such lesions poses a challenge due to difficult nerve accessibility and associated risk of complications [150].

### 13.3. Comparative Features

In animals, extensive research on the rat hypoglossal nucleus using Nissl and Golgi techniques has been conducted. These techniques have revealed that neurons within the nucleus can be classified into distinct and recognizable sets. Such categorization has been established based on specific, detailed, objective criteria. The extent to which the structural features of hypoglossal neurons, including their size, shape, neuronal density, and variable dendritic arborization patterns, along with the adaptability of afferent field terminals, can influence intricate and modulated tongue movements within the framework of the established principles of neuromuscular motor mechanisms remains a subject of debate [151]. In dogs, rabbits, and rats, hypoglossal motor neurons were primarily categorized into two principal groups: ventral (or medial) and dorsal (or lateral). Axons from the ventral group traverse through the medial branch of the hypoglossal nerve, whereas those from the dorsal group pass through its lateral branch. Motor neurons innervating the geniohyoid or genioglossus muscle are localized on the ventral or lateral face of the ventral group. Conversely, hypoglossal and styloglossal motor neurons are situated on the lateral face of the dorsal group [152].

Using the horseradish peroxidase (HRP) method, the cytological architecture of hypoglossal nuclei in macaque monkeys has been extensively analyzed. At rostral levels, the hypoglossal nuclei could be segmented into medial and lateral divisions. At caudal levels, they were subdivided into mediodorsal, medioventral, lateral, ventral, and laterodorsal divisions. HRP experiments revealed that motor neurons in the medial, mediodorsal, medioventral, and ventral divisions project their axons to the medial branch, whereas those in the lateral and laterodorsal divisions innervate the lateral branch. It appears that the motor neurons responsible for controlling the muscles that protrude the tongue are situated more centrally or lower within the hypoglossal nucleus compared to those controlling the muscles responsible for retracting the tongue. In macaques, the arrangement within the hypoglossal nucleus follows a topographical pattern like that observed in cats. However, motor neurons controlling the genioglossus muscle are distributed more dorsally in monkeys compared to cats.

A study performed a dissection for adult *Elaphe obsoleta quadrivittata* and microscopic examination of serial sections for late embryos of *Thamnophis ordinoides*. The cranial nerves exhibited similarities to those typically found in lizards. The hypoglossal nerve displayed considerable size and a relatively short but intricate course as it medially approached tongue musculature. It enters the muscle mass just anterior to the raphe, marking the anterior boundary of the hyoglossus muscles. Subsequently, one or two minor nerves emerge to provide innervation to the neurocostomandibular muscle complex.

## 14. Conclusions

The study of cranial nerves is of paramount importance in the realms of both clinical medicine and neuroscience. These twelve cranial nerves, with their specific functions and intricate pathways, serve as a foundational framework for understanding the human nervous system. Through meticulous study and clinical observations, researchers and healthcare professionals can identify, diagnose, and treat an array of neurological conditions and disorders, ranging from motor neuron diseases to cranial nerve injuries. Furthermore, insights into the structural and functional diversity of these nerves are pivotal for advancing our understanding of neuroanatomy, contributing to ongoing neuroscience research, as they are central for cognitive and physiological functions. Thus, further research is needed to elaborate upon these notes on cranial nerves.

## Figures and Tables

**Table 1:** The cranial nerves in brief.

Cranial Nerve Number	Name	Type	Function
I	Olfactory	Sensory	Smell (detection of odors)
II	Optic	Sensory	Vision (the “seeing” process)
III	Oculomotor	Motor	Eye movement, pupil control, eyelid elevation
IV	Trochlear	Motor	Coordination of binocular vision
V	Trigeminal	Mixed	Sensory (as painful) and motor (as mastication) to the face
VI	Abducens	Motor	Lateral eye movement (abduction)
VII	Facial	Mixed	Facial expression, eyelid and lips closure, taste in the anterior 2/3 of the tongue
VIII	Vestibulocochlear	Sensory	Hearing, balance, and equilibrium
IX	Glossopharyngeal	Mixed	Taste perception, swallowing, specific blood pressure
X	Vagus	Mixed	Multiple bodily functions such as speech, digestion, respiration, cardiac activity, and possibly the immune system
XI	Accessory	Motor	Head, neck, shoulder movement
XII	Hypoglossal	Motor	Tongue movement and speech articulation
